# Encoding in Balanced Networks: Revisiting Spike Patterns and Chaos in Stimulus-Driven Systems

**DOI:** 10.1371/journal.pcbi.1005258

**Published:** 2016-12-14

**Authors:** Guillaume Lajoie, Kevin K. Lin, Jean-Philippe Thivierge, Eric Shea-Brown

**Affiliations:** 1 University of Washington Institute for Neuroengineering, University of Washington, Seattle, Washington, United States of America; 2 Department of Nonlinear Dynamics, Max Planck Institute for Dynamics and Self-Organization, Göttingen, Germany; 3 School of Mathematics, University of Arizona, Tucson, Arizona, United States of America; 4 School of Psychology, University of Ottawa, Ottawa, Ontario, Canada; 5 Department of Applied Mathematics, University of Washington, Seattle, Washington, United States of America; 6 Department of Physiology and Biophysics, University of Washington, Seattle, Washington, United States of America; University of Pittsburgh, UNITED STATES

## Abstract

Highly connected recurrent neural networks often produce chaotic dynamics, meaning their precise activity is sensitive to small perturbations. What are the consequences of chaos for how such networks encode streams of temporal stimuli? On the one hand, chaos is a strong source of randomness, suggesting that small changes in stimuli will be obscured by intrinsically generated variability. On the other hand, recent work shows that the type of chaos that occurs in spiking networks can have a surprisingly low-dimensional structure, suggesting that there may be room for fine stimulus features to be precisely resolved. Here we show that strongly chaotic networks produce patterned spikes that reliably encode time-dependent stimuli: using a decoder sensitive to spike times on timescales of 10’s of ms, one can easily distinguish responses to very similar inputs. Moreover, recurrence serves to distribute signals throughout chaotic networks so that small groups of cells can encode substantial information about signals arriving elsewhere. A conclusion is that the presence of strong chaos in recurrent networks need not exclude precise encoding of temporal stimuli via spike patterns.

## Author Summary

Recurrently connected populations of excitatory and inhibitory neurons found in cortex are known to produce rich and irregular spiking activity, with complex trial-to-trial variability in response to input stimuli. Many theoretical studies found this firing regime to be associated with chaos, where tiny perturbations explode to impact subsequent neural activity. As a result, the precise spiking patterns produced by such networks would be expected to be too fragile to carry any valuable information about stimuli, since inevitable sources of noise such as synaptic failure or ion channel fluctuations would be amplified by chaotic dynamics on repeated trials. In this article we revisit the implications of chaos in input-driven networks and directly measure its impact on evoked population spike patterns. We find that chaotic network dynamics can, in fact, produce highly patterned spiking activity which can be used by a simple decoder to perform input-classification tasks. This can be explained by the presence of low-dimensional, input-specific chaotic attractors, leading to a form of trial-to-trial variability that is intermittent, rather than uniformly random. We propose that chaos is a manageable by-product of recurrent connectivity, which serves to efficiently distribute information about stimuli throughout a network.

## Introduction

Highly recurrent connectivity occurs throughout the brain. It is believed that recurrent cortical circuits typically operate in a “balanced state” in which every neuron receives a large number of excitatory (E) and inhibitory (I) inputs (see, e.g., [1–3]). This means synaptic currents nearly cancel on average, but feature strong fluctuations, giving rise to sustained irregular spiking [4]. Well-established results show that such strongly recurrent networks operating in a balanced regime can produce *chaotic* dynamics in a range of settings, from abstract firing rate models with random connectivity [5] to networks of spiking units with excitatory and inhibitory cell classes [2, 6–8]. Chaos implies that the network dynamics depend very sensitively on network states, so that tiny perturbations to initial conditions may lead to large effects over time. As a consequence, when the same stimulus is presented to a chaotic network multiple times, it may fail to generate reproducible responses. How can stimuli be encoded in the variable spike trains that result (c.f. [9, 10])? A central issue is the relationship between trial-to-trial variability and input discriminability: since exactly the same sensory input can elicit different neural responses from one trial to the next (as can distinct inputs), how can one decide which stimulus is driving a network based on its response?

**Fig 1:**
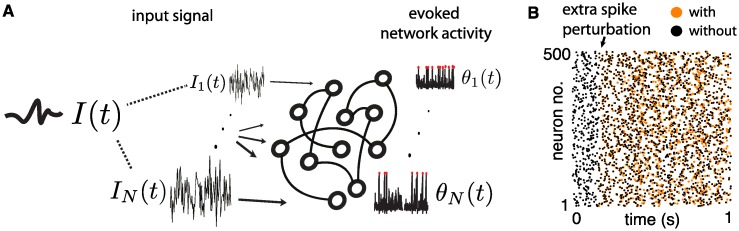
(**A**) An input *I*(*t*), with *N* independent components, is presented to a chaotic network of *N* spiking cells. (**B**) Raster plot of the network response to a fixed input *I*(*t*), on two trials. Arrow indicates a perturbation to the network on the second trial, in which an extra spike is “added” to neuron number 1. Different color markers indicate the widely divergent spike rasters that occur with and without the perturbation.

To illustrate the variable stimulus responses due to chaos, Fig 1 shows a model balanced network (to be described in detail below) driven by a fixed multi-dimensional stimulus, as well as a raster plot of the spiking output of the network on two trials. In each of these trials, the exact same stimulus is presented, but on the second trial a small perturbation is artificially introduced in the form of an extra spike for neuron 1 (as in [11–13]). This small perturbation quickly reverberates throughout the whole network in a seemingly random fashion [11–13]. Can a network this sensitive to small differences in its internal state produce sufficiently reliable outputs for discrimination? If we were to present two different stimuli to such a network, could a decoder be trained to discriminate the spikes evoked by the first input from the spikes evoked by the second, despite both sets of output spikes being subject to the type of variability shown here? This is the central question that we investigate here.

We study this stimulus coding question using a strongly chaotic, recurrent spiking network model driven by temporal stimuli. The strength of stimulus inputs is comparable to network interactions, so that dynamics are not dominated by external stimuli alone. We find that, despite chaos, the network’s spike patterns encode temporal features of stimuli with sufficient precision so that the responses to close-by stimuli can be accurately discriminated. We relate this coding precision to previous work grounded in the mathematical theory of dynamical systems, which shows that—at the level of multi-neuron spike patterns—chaotic networks do not produce as much variability as one might guess at first glance [14, 15]. This is because in such networks, the trial-to-trial variability of spike trains evoked by time-dependent stimuli leads to the formation of low-dimensional chaotic attractors. Our main findings are:

1. It is possible for strongly chaotic recurrent networks to produce multi-cell spike responses that remain discriminable even for highly similar temporal inputs.
2. The same recurrent connections that produce chaos distribute stimulus information throughout the network, so that stimuli can be discriminated based on only a small subset of “readout” neurons.
3. Input statistics influence the strength of chaotic fluctuations that can obscure stimuli. We quantify this via a chaos-induced “noise floor”; stimuli whose strength exceeds this floor will be easily discriminable.

We show that these findings are consistent with well-understood properties of irregular activity produced in balanced networks [2, 5, 16]. We also demonstrate that classical mean-field theories describe the overall firing statistics in our networks, but that additional tools are needed to describe their response to temporal stimuli.

Our results are based on numerical simulations guided by mathematical theory, but connect to a broad experimental literature: trial-to-trial variability in neural responses to repeated stimuli is often observed empirically [1, 17–19] (though see also [20–24]). Even though there are many likely contributors to this variability, ranging from stochasticity in neurotransmitter release or ion channels ([25], but see [26, 27]) to confounding factors like behavioral state, activity level, and levels of adaptation [28, 29], chaotic interactions may play an inescapable role. Indeed, chaos may represent a mechanism by which other sources of variability are amplified. Importantly, we find that chaos in driven networks produce a type of intermittent variability, with some spiking “events” predictably repeated across trials [14, 15] (cf. [30–32]). This is also reported in a number of experiments (see e.g. [33, 34] and [23]).

The remainder of this paper is organized as follows. In the *Methods* section, we describe our network model, the input discrimination task we use throughout, and outline our implementation of the Tempotron classifier [35], which we train on the spikes produced by our network. We also compare the across-trials statistics of our network to more standard mean-field ensembles, showing that fluctuating time-dependent stimuli can induce complex statistical structure across trials. In the *Results* sections, we first present an analysis of single neuron statistics in our network. This is followed by an overview of dynamical systems concepts useful to describe chaotic dynamics, which we use to interpret the spike outputs our model produces. Finally, we present our main findings based on the performance of trained classifiers, offering explanations of underlying mechanisms at the level of dynamical network interactions.

## Methods

We study a recurrent network of excitatory and inhibitory neurons with random, sparse coupling, as in [2, 3, 6, 14]. Every neuron *i* = 1, …, *N* in our network receives an external input signal *I*_*i*_(*t*), which we describe in more detail below. The collection of all these signals is an *N*-dimensional input that we denote by *I*(*t*) = {*I*_*i*_(*t*)}_*i* = 1,…,*N*_ and simply call the *network’s input* or *stimulus* (see Fig 1 (A)). We emphasize that the inputs have *N* independently varying components; i.e., they are *N*-dimensional.

Our goal is to explore the ability of a decoder to discriminate between two distinct network inputs *I*^*A*^(*t*) and *I*^*B*^(*t*), based on the activity of the network (or of a subset of cells from the network). We now describe the model in detail, as well as the specific discrimination task and decoding paradigm we use.

### Model

We consider a network of *N* neurons separated into excitatory (E) and inhibitory (I) populations of sizes *N*_*E*_ and *N*_*I*_ obeying “Dale’s Law” [36] (i.e. neuron can either have all inhibitory or all excitatory outgoing synapses). We set *N*_*I*_ = 0.2*N*, *N*_*E*_ = 0.8*N* and couple the network according to the random and sparse, *balanced state* architecture [2, 3, 6, 14, 37] following a standard Erdös-Rényi scheme with mean in-degree *K* ≪ *N*. This means a synaptic connection from a neuron *j* to a neuron *i* is drawn randomly and independently with probability *K*/*N*_E,I_ where E or I denotes the type of neuron *j*. Throughout the paper, we report simulations carried out with *N* in a range from 500 to 5000 and *K* from 10 to 500 but note that the majority of the results we outline are independent of network size, or scale with *N* in simple ways while *K* plays a more subtle role that does not impact the qualitative nature of our results.

Individual neurons are modelled as Quadratic-Integrate-and-Fire (QIF) units [38]. The state of each neuron *i* = 1, …, *N* is represented by a voltage variable *v*_*i*_ ∈ (−∞, ∞). These voltages evolve according to intrinsic voltage-dependent (QIF) dynamics

$$\tau_{m}\frac{dv_{i}}{dt}=\frac{\left(v_{i}-v_{R}\right)\left(v_{i}-v_{T}\right)}{\Delta v}+I_{syn}\left(t\right)$$ (1)

where *τ*_*m*_ is the membrane time-constant, *v*_*R*_ and *v*_*T*_ are rest and threshold potentials, Δ*v* = *v*_*T*_ − *v*_*R*_ and *I*_*syn*_(*t*) represents incoming inputs to the neurons, both from the network and from our external stimulus. The dynamics of Eq (1) are discontinuous in time: once the membrane potential *v* exceeds the threshold, it blows up to infinity in finite time at which point a spike is said to be emitted and *v* is manually reset to −∞. For convenience, we use *v*_*T*_ = −*v*_*R*_ = 1 and apply a smooth change of coordinates *v*(*θ*) = tan(2*πθ* − *π*)/2 that maps these unbounded values to phase variables *θ*_*i*_ ∈ [0, 1]. QIF dynamics acting in these coordinates is known as the *θ*-*neuron* model [38]. Here *θ*_*i*_ = 0 and *θ*_*i*_ = 1 represent the same state of *v*_*i*_ = ±∞, and a neuron is said to “spike” once it reaches this point. Mathematically, this means that the voltage of each neuron is represented by a point on a circle and the state of the entire network at time *t* is given by the vector of phases *θ*(*t*) = (*θ*_1_(*t*), …, *θ*_*N*_(*t*)). For the sake of clarity, we report spikes and other temporal observables using milliseconds by fixing the neural time constant *τ*_*m*_ = 10 ms in the QIF coordinates and rescaling unit-less time by 2*πτ*_*m*_ (see [38] for more details about this coordinates change).

Thus, the state of the network as a whole can be thought of as a moving point on an *N*-torus ($T^{N}$). The dynamics of each neuron –representing an axis on the torus– is given by

$${\dot{\theta }}_{i}=F\left(\theta_{i}\right)+Z\left(\theta_{i}\right)\sum_{j=1}^{N}a_{ij}g\left(\theta_{j}\right)+Z\left(\theta_{i}\right)\underset{I_{i}\left(t\right)}{\underset{︸}{\left[\eta +\varepsilon \xi_{i}\left(t\right)\right]}}$$ (2)

where *F*(*θ*_*i*_) = 1 + cos(2*πθ*_*i*_), *Z*(*θ*_*i*_) = 1 − cos(2*πθ*_*i*_) (the canonical phase response curve of a Type I neuron [39]), and *g*(*θ*_*j*_) is a sharp “bump” function, nonzero only near the spiking phase *θ*_*j*_ = 1 ∼ 0. As in [14, 15], we set

$$g\left(\theta \right)=\left\{\begin{array}{cc} d{\left(b^{2}-{\left[\left(\theta +\frac{1}{2}\right)\text{mod}\;\;1-\frac{1}{2}\right]}^{2}\right)}^{3} & \text{;}\;\theta \in [-b,b]\\ 0 & \text{;}\;\text{else} \end{array}\right.$$

with *b* = 1/20 and *d* = 35/32. This phase coupling function is chosen to model the rapid rise and fall of post-synaptic currents, while being differentiable everywhere so that the vector field defined by Eq (2) remains smooth. Note also that $\int_{0}^{1}d\theta g\left(\theta \right)=1$. The synaptic weight *a*_*ij*_ from neuron *j* to neuron *i*, if non-zero, is set to a fixed value that depends on both neuron types. Abusing notation slightly, we set: $a_{\text{EE}}=a_{\text{IE}}=\alpha /\sqrt{K}$, $a_{\text{EI}}=-\alpha /\sqrt{K}$ and $a_{\text{II}}=-\rho \alpha /\sqrt{K}$. We set *α* = 0.35 and *ρ* = 0.75 so that I-neurons are slightly less inhibited by other I-neurons than E-neurons, as in the original balanced state architecture [2]. The term *I*_*i*_(*t*) represents an external input stimulus to neuron *i*; here modelled by the sum of a DC current *η* and independent Gaussian white-noise processes *ξ*_*i*_(*t*) scaled by *ε*. We note that *η* can take negative values which places neurons in an excitable regime [38]. Both *η* and *ε* are global parameters that are fixed across all neurons. Throughout most of the paper, they are set to *ε* = 0.5, *η* = −0.5 so that the network is fluctuation-driven, producing sustained irregular activity characterized by a broad firing rate distribution with a mean roughly between 10 and 20 Hz. Together, the input signals to each neuron form the global input to the network *I*(*t*) = (*I*_1_(*t*), …, *I*_*N*_(*t*)) as depicted in Fig 1 (A). We require that the realizations *I*_*i*_(*t*) be statistically independent from each other across neurons *i*, so that the network’s input *I*(*t*) = {*I*_*i*_(*t*)}_*i*_ is free of redundancies and can be thought of as a *N*-dimensional signal, but we briefly address the implication of such correlations in the Discussion section.

We stress that *I*(*t*) models inputs to the system, and not the various molecular and cellular sources of noise associated with neuronal dynamics. We give more details about the statistics of *I*(*t*) below, but note that in this regime and all other considered in this paper, we have verified that our network is chaotic by showing the presence of positive Lyapunov exponents, a standard measure for sensitivity to initial conditions (see [14, 15] for more details).

#### Numerical simulation details

A standard Euler-Maruyama scheme [40] was used to numerically integrate Eq (2), treated as a stochastic differential equation (SDE). Because of the state-dependent factor multiplying white noise, multiple interpretations of the SDE are possible (see, e.g., [41] for an explanation). Following [42], we interpret our equation as a Stratonovich SDE. Accordingly, a correction term $\frac{\varepsilon^{2}}{2}Z\left(\theta_{i}\right)Z^{\prime }\left(\theta_{i}\right)\Delta t$ is added to the right hand side of the discretized equation [40]. A time-step of Δ*t* = 0.05 ms was used for all simulations. We verified that smaller temporal resolution did not change our results. For estimates involving sampling of many trajectories within response-ensembles, initial states of the network were uniformly sampled over state space $T^{N}$. Numerical estimates of Lyapunov exponents were obtained by evolving the associated variational equation of Eq (2); further details can be found in [14, 15]. Large batched simulations were carried out on the NSF XSEDE *Science Gateway* supercomputing platform.

Numerical simulations were implemented in the Python and Cython languages. Computations of statistical quantities such as pairwise trajectory distances and spike-time reliability, as well as Tempotron classifier training and testing, were implemented in MATLAB.

### Discrimination task and response ensembles

The stimulus *I*(*t*) = (*I*_1_(*t*), …, *I*_*N*_(*t*)) mimics a collection of highly featured temporal inputs to each neuron in the network. In this framework, the response of the network to a specific input can be modelled by “freezing,” or choosing specific realizations of the stochastic processes *ξ*_*i*_(*t*), the fluctuating component of *I*_*i*_(*t*); this is sometimes known as a “frozen noise” experiment [26, 27]. We emphasize that even though input components *I*_*i*_(*t*)’s are modelled as random processes, they represent signals impinging on the network, rather than various biological sources of noise. In fact, we do not model any biological noise; our model should be viewed as a deterministic, non-autonomous dynamical system. All trial-to-trial variability is generated by chaos which amplifies discrepancies in initial states. These discrepancies are abstracted as randomly sampled initial states and may, in reality, be produced by noise. We revisit this distinction in greater detail in the *Discussion*.

#### Response ensembles

Suppose that an input *I*(*t*) is presented to the network starting at time *t* = 0, and that the initial state of the network at that time is $\theta^{0}=\left(\theta_{1}^{0},\ldots ,\theta_{N}^{0}\right)$ (i.e., the “voltages” of all neurons at *t* = 0). The subsequent evoked trajectory *θ*(*t*) is uniquely defined and depends on both *I*(*t*) and *θ*_0_. However, for a chaotic system like our network, even small changes in *θ*_0_, or even worse, a completely unknown initial state, can lead to big differences in the subsequent trajectories *θ*(*t*). If one of two inputs *I*^*A*^(*t*) and *I*^*B*^(*t*) is presented to the network starting at *t* = 0 and that the initial state is unknown, could a decoder reading out the evoked network activity be trained to tell which input was shown? How similar can the two inputs be before the decoder fails? To answer these questions, an essential concept is that of “response ensembles.”

To begin, consider an ensemble of many network states *θ*^0^. To this ensemble corresponds another one, consisting of network responses, i.e., a version of the network’s activity parametrized by *t* > 0 for each choice of initial state *θ*^0^, in response to the same *I*(*t*). Each “response” in this ensemble represents a different “trial,” much like in an experiment where exactly the same stimulus is repeatedly presented to a system. Trial-to-trial variability thus depends on how distributed these ensembles of responses are about the network’s state space.

**Fig 2:**
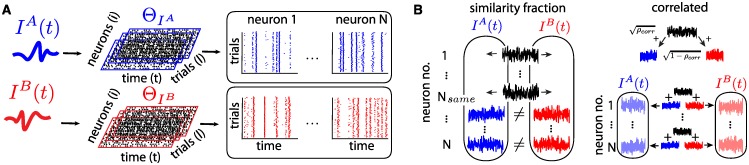
(**A**) Two ensembles of spike patterns are generated by distinct, *N*-dimensional inputs *I*^*A*^ and *I*^*B*^ repeatedly presented to the network on multiple trials, where initial states are chosen at random. This results in two network-wide response ensembles Θ_*I*^*A*^_ and Θ_*I*^*B*^_ containing spike patterns across neurons, time and trials. (**B**) Illustration of the two procedures used to control input similarity. Left: *ρ*_*same*_ controls the number of neurons *N*_*same*_ = *ρ*_*same*_ *N* that receive identical inputs *I*_*i*_(*t*) under both stimuli *I*^*A*^(*t*) and *I*^*B*^(*t*). Right: *correlation coefficient* *ρ*_*corr*_ controls the correlation of all pairs of neural inputs $I_{i}^{A}\left(t\right)$ and $I_{i}^{B}\left(t\right)$ (note that *I*_*i*_(*t*) and *I*_*j*_(*t*) remain uncorrelated if *i* ≠ *j*).

Formally, we define a *response ensemble* associated with an input *I*(*t*) as Θ_*I*_: the collection of network trajectories through state space, in response to *I*(*t*), for which initial states were sampled independently from some initial probability distribution. In this paper, we choose this distribution to be the uniform one, meaning that each point *θ*^0^ in state space has equal probability of being picked. We call individual trajectories within a response ensemble *trials*. A response ensemble is thus indexed by 3 components: neuron number *i* (“space”), time *t*, and trial number *l* as depicted in Fig 2 (A). For example, Θ_*I*_(*i*, *t*, *l*) represents the state of neuron *i* at time *t* on trial *l*, the average across trials 〈Θ(*i*, *t*)〉_*l*_ represents the peristimulus time-histogram (PSTH) [43] of neuron *i*, etc. Throughout, we also study the spike patterns associated with the trajectories forming Θ_*I*_ and often refer to the collections of these spike times as response ensembles as well (see Fig 2 (A)).

We note that the response ensembles defined here differ fundamentally from the statistical ensembles that underlie the *mean-field* (MF) theories often used in mathematical neuroscience (see e.g. [2, 16]). While response ensembles are defined for a given, specific stimulus history *I*(*t*) (so that for each stimulus realization there is, in principle, a different ensemble), the statistical ensembles used in those other theories typically consist of trajectories generated by both random initial conditions *and* independent, random stimuli. As such, those ensembles cannot be used to model the type of discrimination task we are interested in here. In more probabilistic language, equating “ensembles” with stochastic processes, our response ensembles are precisely the conditional probability distributions obtained by conditioning a MF-type statistical ensemble by a particular stimulus history *I*(*t*); see, e.g., [44] for a more detailed discussion of response ensembles.

The choice of statistical ensemble can have significant consequences for statistical measures like correlations (between cells and in time): for the same system, correlations computed with MF-type statistical ensembles can differ from those computed with respect to the response ensemble evoked by a specific *I*(*t*). We will return to this distinction in the context of our networks in the *Results* section.

#### Discrimination task

With the concept of response ensembles, we can now give more precise formulations of various questions related to discrimination. First, we ask how much overlap exists between the response ensembles Θ_*I*^*A*^_ and Θ_*I*^*B*^_. This is controlled by two factors: how much state space is occupied by each ensemble at a given time, and how close they are to each other. Second we train a decoder on the spike patterns associated with the network responses. We describe this decoder in the next section. Importantly, when we compare the network’s response to two stimuli, we also require *ε* and *η* to be the same for *I*^*A*^(*t*) and *I*^*B*^(*t*). This way, the averaged statistics of network responses are identical for any stimulus pair, and discrimination must rely on the differences in random fluctuations between the two specific realizations *I*^*A*^(*t*) and *I*^*B*^(*t*).

To study our network’s sensitivity to changes in its inputs, we introduce two notions of the similarity, or “distance”, between the stimuli *I*^*A*^(*t*) and *I*^*B*^(*t*). Fig 2 (B) illustrates the effect of both paradigms. First, we define *ρ*_*same*_ ∈ [0, 1] as the proportion of the network’s neurons that receive identical inputs ($I_{i}^{A}\left(t\right)=I_{i}^{B}\left(t\right)$) under both *A* and *B* paradigms; the remaining fraction 1 − *ρ*_*same*_ receive non-identical, independent inputs ($I_{i}^{A}\left(t\right)\neq I_{i}^{B}\left(t\right)$). Second, we vary the correlation *ρ*_*corr*_ ∈ [0, 1] between input pairs driving each neuron *i*, simultaneously for all *i*. Thus, $I_{i}^{A}\left(t\right)$ and $I_{i}^{B}\left(t\right)$ are jointly gaussian processes, each a realization of white noise, with correlation coefficient equal to *ρ*_*corr*_. Note that the inputs to distinct cells remain independent regardless of stimulus choice (${\langle I_{i}^{A,B}\left(t\right)I_{j}^{A,B}\left(t\right)\rangle }_{t}=0$ for *i* ≠ *j*) and that *ρ*_*corr*_ and *ρ*_*same*_ only control the similarity of inputs to the same neuron across different stimuli. In both cases, *ρ*_*corr*_ = *ρ*_*same*_ = 0 enforces that all pairs $\left(I_{i}^{A}\left(t\right),I_{i}^{B}\left(t\right)\right)$ are independent, whereas if *ρ*_*corr*_ = *ρ*_*same*_ = 1, they are identical.

### The Tempotron and discrimination

Our goal is to discriminate between two statistically identical random stimuli based on the network responses they evoke. In the second part of the paper, we do this by training a classifier on the collections of spike times $t=t_{s}^{i}$ evoked by distinct network inputs *I*^*A*^(*t*) and *I*^*B*^(*t*) on finite time intervals [0, *T*]. There are many machine-learning techniques that can perform this task, but our main criteria for a preferred approach are: (i) it should be a useful metric to compare the encoding performance of our network under different conditions and (ii) it should isolate important spike features for coding (interpretability). We therefore opt for a simple, linear classification approach. The results serve as a lower bound on the classification capacity of the network.

There are several approaches one can use to find a hyperplane that separates sets of points in a high-dimensional space, such as the Support Vector Machine [45] (see [46] in the context of spiking data) or other regression techniques. Here, we use a classification method called the *Tempotron* [35], a gradient-descent approach acting on linear weights of temporal kernels designed to mimic the post-synaptic potentials induced by individual spikes. Importantly, the resulting classification hyperplane corresponds to the threshold of a spiking Linear Integrate-and-Fire (IF) neuron model.

The Tempotron receives vector-valued filtered spike trains *s*(*t*) = {*s*_*i*_(*t*)}_*i* = 1…*N*_ where $s_{i}\left(t\right)=\sum_{t_{s}^{i}}K\left(t-t_{s}^{i}\right)$ and $K\left(t\right)=V_{0}\left[e^{-t/\tau_{1}}-e^{-t/\tau_{2}}\right]$ where *V*_0_ is a normalizing constant. The double-exponential filtering is meant to mimic the rise and fall of synaptic potentials in an IF neuron whose voltage obeys the equation

$$V\left(t\right)=\sum_{i=1}^{N}w_{i}\sum_{t_{s}^{i}}K\left(t-t_{s}^{i}\right)+V_{rest}$$ (3)

with voltage threshold set at *V*_*thr*_ = 1. Thus, the Tempotron computes the sum of the filtered network spike trains, according to the readout weights *w*_*i*_ and the timescale set by its kernel. We tune the filter’s decay and rise time-constants to *τ*_1_ = 20 and *τ*_2_ = 3.75 ms as in [35], to impose an intrinsic sensitivity to spike timing at that resolution.

The Tempotron’s goal is to fire at least one spike (i.e. cross its threshold *V*_*thr*_) when presented with a network spike output associated to *I*^*A*^(*t*) while refraining from firing when the network responds to *I*^*B*^(*t*). Following [35], we train the Tempotron to classify spike outputs on finite-length time intervals [*t*_0_, *t*_0_ + *T*] using a fixed number of trials from $\Theta_{I^{A}}^{t}$ and $\Theta_{I^{B}}^{t}$ respectively, and test the trained classifier on new trials. Thus, beyond discriminating between two “training” ensembles of spikes, we test the ability of the Tempotron to generalize and discriminate new patterns, related to training sets in that they are sampled from the same chaotic response ensemble. The robustness of the Tempotron to Gaussian, random spike-time jitters is well established [35]; here we investigate the effect of chaotic variability of the type generated by our networks.

**Fig 3:**
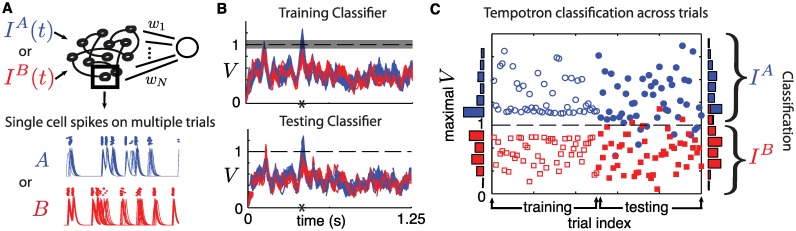
(**A**) Schematic of a Tempotron with readout weights *w*_*i*_. For one neuron from the chaotic network, we show spike times (dots) of a randomly chosen neuron across several trials for inputs *I*^*A*^ or *I*^*B*^, together with the filtered traces for these spike times. (**B**) Tempotron voltage traces *V*(*t*) for a time window of *T* = 1.25 seconds. Top: traces used for training. Bottom: traces used for testing. Grey shaded area surrounding threshold *V*_*thr*_ shows training margin. Asterisk shows time of maximal output. (**C**) Classification for all training and testing trials. Markers show the Tempotron’s maximal voltage *V* in time window *T*. A correct classification corresponds to blue circles (*I*^*A*^) above threshold and red squares (*I*^*B*^) below. For all panels, the stimuli *I*^*A*^ or *I*^*B*^ have *ρ*_*same*_ = 0.9.

Fig 3 illustrates the process. Out of 100 trials from each ensemble, we select 50 from Θ_*I*^*A*^_ and 50 from Θ_*I*^*B*^_ to train the readout weights *w*_*i*_ in Eq (3). Fig 3 (A) illustrates the filtered spike output of a randomly selected neuron on the training trials from *I*^*A*^ and *I*^*B*^. The remaining trials will be used for testing. We employ the algorithm described in [35] to find a set of weights *w*_*i*_ imposing that the voltage *V* of the Tempotron will exceed the threshold *V*_*thr*_ = 1 at least once in the pattern time-window when presented with spikes from a trial from $\Theta_{I^{A}}^{t}$ while remaining sub-threshold when receiving spikes from Θ_*I*^*B*^_. We report results for a time window of *T* = 1.25 seconds. We find that training often fails for *T* < 50 ms, but that the results we present below remain qualitatively unchanged for bigger *T*. We use a margin of *V*_*thr*_ ± 0.1 in the training to ensure a good separation (c.f. [35]). Fig 3 (B) shows the output of the Tempotron during training and testing while panel (C) compares the training and testing outcomes by showing the maximal *V* within the *T*-window. In this example, even though inputs are quite similar (*ρ*_*same*_ = 0.9), only a few test trials are misclassified.

To quantify the discriminability of spike patterns, we define the *performance* *P* as the fraction of successful test classifications. Note that *P* has a maximal value of 1 and a minimal value of 0.5 which corresponds to chance. We average *P* by retraining the Tempotron 20 times using different training and testing trials from our ensembles. As an example, the performance *P* of the classification in Fig 3 (C) is about 0.9.

### Asynchronous activity and mean-field approximations

Our network has basic statistical features expected from prior work on sparse balanced networks: the activity of neurons is mostly decorrelated in time and between cells, and is statistically stationary (see e.g. [2, 16], but also our note on “correlations” below). These features are useful in deriving simplified expressions for neuronal population temporal statistics. In *mean-field* (MF) theory, one replaces a complex network of interacting units with a reduced model of a single unit driven by a relatively simple independent stochastic process, meant to model the outputs of all other units within the network impinging on the given unit. We now follow this approach to (1) derive an analytic expression for E and I population firing rates and fluctuation amplitudes, and (2) demonstrate that our network operates in an asynchronous balanced regime that is consistent with prior work. The results of this section will help us design surrogate population dynamics—used throughout the paper—to compare chaotic dynamics to basic assumptions of independent noise.

A note about our use of the term “correlation:” as mentioned earlier, even for the same system, correlations depend on the choice of statistical ensemble. Because the term has many potentially different meanings, we have strived to be as explicit as we reasonably can in what follows. In this section, we are concerned with correlations averaged across independent stimulus realizations, as is the custom in MF theory. The nature of correlations across trials in our network is revisited in *Results*, and is treated in detail in [14, 15].

We follow a similar approach to [47, 48] where QIF neurons driven by noise and in a MF setting are studied for exponentially decaying synapses. In contrast to networks of leaky integrate-and-fire (LIF) neurons, QIF dynamics introduce some complications because their firing rate response to fluctuating drives is not straightforward to compute (c.f. [48–50]). Here we follow the general approach of [16] using a number of simplifications to arrive at an estimate for the mean firing rate or neurons in our network.

We begin by re-writing Eq (2) as

$$\frac{d\theta_{i}^{\Omega }}{dt}=F\left(\theta_{i}^{\Omega }\right)+Z\left(\theta_{i}^{\Omega }\right)\left[I_{net}^{\Omega }\left(t\right)+I_{i}\left(t\right)\right]$$ (4)

with Ω denoting neuron type (E or I) and

$$\begin{array}{cc} I_{net}^{\Omega }\left(t\right) & =\sum_{j=1}^{N}a_{ij}g\left(\theta_{j}\left(t\right)\right)\\ I_{i}\left(t\right) & =\eta +\varepsilon \xi_{i}\left(t\right). \end{array}$$

**Fig 4:**
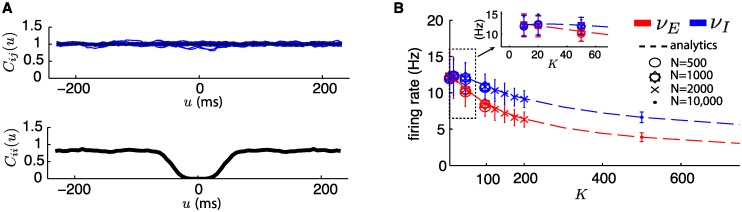
(**A**) Top: Typical normalized spike-time cross-correlogram *C*_*ij*_(*u*): expected proportion of pairs of spikes separated by *u* ms, for random pairs of neurons within the network. In blue: for 30 individual pairs; in black: average. Bottom: Typical normalized spike-time auto-correlogram *C*_*ii*_(*u*) for a neuron in the network. The QIF dynamics impose a relative refractory period close to spiking which gives a departure from purely Poisson firing statistics (notice the dip toward time 0). Typical Fano Factors are between 0.77 and 1. For A and B, *N* = 500, *K* = 20; however, the shape of these functions is not affected by *N* or *K*. Statistics sampled over network simulations of 300 seconds. (**B**) Mean firing rate of E and I neurons within the network as a function of *K*. Dotted line shows MF estimate (see Eq 8). Various markers show estimates sampled from all neurons in simulated networks of different sizes *N*. Error bars show one standard deviation. Inset shows zoom for low-*K* values.

Since connectivity is sparse and *K*, *N* are large, we assume that (i) Pre-synaptic spike trains to a neuron are statistically independent and (ii) Each spike train is Poisson distributed with constant rate *ν*_*E*_ or *ν*_*I*_. Assumption (i) is justified by the flat spike-time cross-correlograms observed across pairs of neurons in the network, shown in the top panel of Fig 4 (A). This holds for networks as small as *N* = 500 and in-degree as small as *K* = 10. Assumption (ii) on the other hand is not quite met, as shown by the dip in the typical spike-time auto-correlogram of a neuron in the bottom panel of the same figure. This dip occurs because QIF dynamics produce a relative refractory period, leading to typical Fano Factors lower than one (between 0.77 and 1 in all of our simulations). This is refractory period can be regarded as a realistic feature; in any case, the use of assumption (ii) still leads to correct estimates for our dynamical regime, as was also observed in [47].

Since $\frac{d\theta }{dt}{|}_{\theta =1}=2$, we make the approximation $\int dtg\left(\theta_{j}\left(t\right)\right)=\frac{1}{2}\int d\theta_{j}g\left(\theta_{j}\right)≃\frac{1}{2}\int d\theta_{j}\delta \left(\theta_{j}-1\right)$. Thus, the mean and variance of the compounded network input $I_{net}^{\Omega }\left(t\right)$ over a small time interval Δ*t* are approximated by *K*(*a*_Ω*E*_ *ν*_*E*_ − *a*_Ω*I*_ *ν*_*I*_)Δ*t*/2 and $K(a_{\Omega E}^{2}\nu_{E}+a_{\Omega I}^{2}\nu_{I})\Delta t/4$ respectively. It follows that the dynamics of a typical neuron of type Ω can be represented by the following stochastic differential equation (SDE) [16]

$$\frac{d\theta_{i}^{\Omega }}{dt}=F\left(\theta_{i}^{\Omega }\right)+Z\left(\theta_{i}^{\Omega }\right)\left[\underset{∼I_{net}^{\Omega }\left(t\right)}{\underset{︸}{\mu_{\Omega }+\sigma_{\Omega }\zeta \left(t\right)}}+\underset{I_{i}\left(t\right)}{\underset{︸}{\eta +\varepsilon \xi (t)}}\right]$$ (5)

with

$$\begin{array}{cc} \mu_{E} & =\frac{\alpha }{2}\sqrt{K}\left(\nu_{E}-\nu_{I}\right)\\ \mu_{I} & =\frac{\alpha }{2}\sqrt{K}\left(\nu_{E}-\rho \nu_{I}\right)\\ \sigma_{E}^{2} & =\frac{\alpha^{2}}{4}\left(\nu_{E}+\nu_{I}\right)\\ \sigma_{I}^{2} & =\frac{\alpha^{2}}{4}\left(\nu_{E}+\rho^{2}\nu_{I}\right) \end{array}$$ (6)

where *ζ*(*t*) and *ξ*(*t*) are independent Gaussian white noise processes with zero mean and unit variance. We can combine both input terms $I_{net}^{\Omega }\left(t\right)$ and *I*_*i*_(*t*) in Eq (5) to get an SDE with a single stochastic term:

$$\frac{d\theta_{i}^{\Omega }}{dt}=F\left(\theta_{i}^{\Omega }\right)+Z\left(\theta_{i}^{\Omega }\right)\left[\left(\mu_{\Omega }+\eta \right)+\sqrt{\sigma_{\Omega }^{2}+\varepsilon^{2}}\zeta \left(t\right)\right].$$ (7)

Here, the combination of network interactions and of the external input signal into one noise term is made possible by the fact that mean E and I firing rates do not depend on any specific realization of input *I*_*i*_(*t*), as long as its mean and fluctuation strength are given by the fixed parameters *η* and *ε*. Thus, just like network interactions, we approximate the effect of any external input by stochastic noise. We show later that for a fixed input stimulus *I*_*i*_(*t*) across trials, as for “frozen noise” experiments (c.f. [51, 52]), both stochastic approximations about network and external inputs fail, because important statistical dependencies are introduced across trials.

Using Equation (3.22) from [48] and the standard change of coordinates from *θ* to *v* and time rescaling described above, we obtain:

$${\bar{\nu }}_{\Omega }=\frac{1}{\tau_{m}}{\left[\int_{-\infty }^{\infty }\frac{dx}{\sqrt{\pi }}\exp\left(-\left(\beta_{\Omega }-\frac{\tau_{s}}{\tau_{m}}\pi \gamma_{\Omega }^{2}\right)x^{2}-\frac{\pi^{2}\gamma_{\Omega }^{4}x^{6}}{12}\right)\right]}^{-1}$$ (8)

where *β*_Ω_ = *μ*_Ω_ + *η*, $\gamma_{\Omega }^{2}=\sigma_{\Omega }^{2}+\varepsilon^{2}$, *τ*_*m*_ is a membrane time-constant and *τ*_*s*_ is a synaptic time constant for exponential synapses. We choose *τ*_*m*_ = 10 ms and take *τ*_*s*_ → 0 as we already approximated our synaptic interactions with *δ*-pulse coupling. This gives time units of ms to the self-consistency Eq (8). We solve for *ν*_*E*_ and *ν*_*I*_ numerically using a function-minimization algorithm (“fminsearch” in MATLAB).

Fig 4 (B) shows that the mean field approximation for the firing rates of neurons in the network matches simulations with great precision for a wide range of *N* and *K*, and that the average firing rates are independent of *N* and change monotonically with *K*, as expected from previous work [16, 47]. Later, we will make use of the quantities *μ*_*E*,*I*_ and $\sigma_{E,I}^{2}$ derived in Eq (6) to outline the difference between network and MF responses for fixed stimuli.

## Results

We now discuss stimulus discrimination based on the output of our network. We begin by investigating the role of network interactions in shaping the statistics of trial-to-trial population responses, comparing these to a simplified model of network dynamics that follows a mean-field approach. We then offer a state-space view of the mechanisms responsible for the observed statistics from the perspective of random dynamical systems. From there, we use spike pattern classification to investigate stimulus encoding in the presence of chaos and formulate our main findings.

### Network interactions are essential for response reliability

We begin by illustrating the statistical structure that emerges across trials when our networks are driven by fluctuating stimuli. Previous work has shown that basic statistical measures such as “noise correlations” may not adequately capture across-trial statistics in driven systems [15]. Here, we follow [14, 15] and take a different approach, comparing the spike patterns produced by our network to those produced by a surrogate spiking model, based on the reduced MF approximation from the *Methods*. Our approach is very similar to that in Fig. 4c of [14], though our comparison here is more extensive and employs quantities derived from MF. Our overall goal is to separate the entraining effect of a fixed stimulus *I*(*t*) from the variability caused by network interactions, or a MF approximation of them.

**Fig 5:**
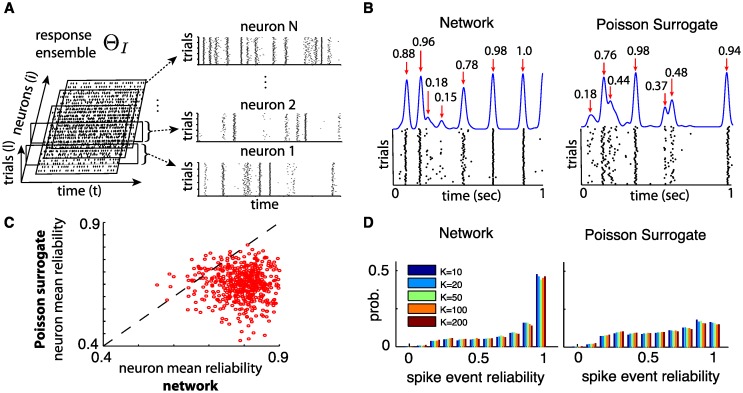
(**A**) Spike patterns from a response ensemble, Θ_*I*_, containing network responses to input *I*(*t*) on many trials. (**B**) Single neuron spike response examples across 50 trials. Left: from a randomly chosen neuron in the network. Right: same neuron as right but replacing network interactions by Poisson spike surrogates following MF assumptions (see text for details). Bottom row shows spike raster plots and top row shows corresponding Gaussian-filtered PSTH (*σ* = 10 ms). Red arrows indicate spike events and numbers indicate spike event reliability. See text for details about calculation of reliability. (**C**) Scatter plot showing the mean reliability of each neuron in a network (*N* = 500, *K* = 20) against its counterpart in the Poisson surrogate inputs paradigm. Neuron mean reliability is the average reliability of all spike events produced by the neuron. (**D**) Left: Histogram of spike event reliability sampled over all spike events produced by networks with different values of *K* (*N* = 2000). Right: Same as left for Poisson surrogate inputs paradigm. For (C) and (D), quantities sampled from simulations lasting 100 seconds over 100 trials.

To do so, it is convenient to recall the response ensembles introduced in *Methods* and depicted in Fig 5 (A): Θ_*I*_(*i*, *t*, *l*). In the MF Eq (8), the mean firing rates *ν*_*E*_ and *ν*_*I*_ represent averages over all indices of response ensembles, 〈Θ_*I*_〉_*i*,*t*,*l*_, and are used as parameters of a Poisson process. Let us define surrogate response ensembles: ${\tilde{\Theta }}_{I}\left(i,t,l\right)$, which are generated numerically in the same way we do for our network (see Methods), except that we replace the interactions from network coupling with Poisson spike trains with homogeneous firing rates *ν*_*E*_ and *ν*_*I*_ given by Eq (8). Note that the same input *I*(*t*) is presented to the *N* neurons in this surrogate “disconnected” network as is presented to the original chaotic network. We verified that the firing rates’ mean and variance are conserved, evidence that ${\langle \Theta_{I}\rangle }_{i,t,l}={\langle {\tilde{\Theta }}_{I}\rangle }_{i,t,l}$, as expected.

For our network, it is clear from Fig 5 (A) that some spikes are repeated across all trials (*reliable* spikes) and others are not (*unreliable* spikes). We return to the mechanisms leading to this intermittent variability in the next section. For now, we ask: how much of this spike time reliability will be conserved in our surrogate response ensemble? We quantify the *reliability* of a spike by estimating the probability of it being repeated on other trials. This is done by convolving the cross-trial spike trains of a neuron with a gaussian filter (standard deviation 10 ms) and adding the resulting waveforms from 100 trials to obtain a filtered peri-stimulus time histogram (PSTH) as illustrated in Fig 5 (B). We call each peak in this histogram a *spike event* and a spike is assigned to an event if it falls within a tolerance time-window defined by the width of the peak at half height [14, 53]. The event’s *reliability* is then estimated by the number of member spikes divided by the number of trials considered. If a spike event has a reliability of 1, it means that one can expect a spike from that neuron at that moment on every trial while a lower reliability indicates more variability. Fig 5 (B) shows examples of spike events with their computed *reliability*, for a randomly chosen neuron in the network and in the Poisson surrogate system.

To quantify the reliability of individual neurons over the course of 100 seconds of activity, we compute their mean reliability, taken to be the average *reliability* of all spiking events they produce. Fig 5 (C) compares the mean reliability of neurons during network responses and in the presence of surrogate inputs. It is clear that most neurons are significantly more reliable when network interactions are present, as opposed to independent stochastic inputs. To access the overall reliability of population-wide response ensembles, we estimate the distribution of spiking event *reliability* over all neurons. Fig 5 (D) shows histograms obtained by sampling all spike events in the network, together with surrogate ensembles. Here we take *N* = 2000 and multiple values of *K*, simulated for 10 seconds over 100 trials. A number of features can be observed from these plots: (i) Despite chaotic dynamics, a majority of spike events in driven networks are reliable (as in [14]). (ii) There are substantially more reliable spikes in networks than in surrogate populations (cf. [14, 15]). (iii) Spike reliability does not depend on the connectivity in-degree *K*.

Important conclusions can be drawn from these observations. First, as shown in [14, 15], MF-like approximations of network interactions are insufficient to capture the statistical properties of trial-to-trial variability, and actually over-estimate it. Cross-trial statistics in chaotic networks show population-wide dependencies despite independent temporal statistics, a feature that is lost by averaging network interactions.

Second, it shows that reliable spikes observed in chaotic networks are not solely due to strong driving inputs. Such reliability could indeed be generated if inputs *I*_*i*_(*t*) had fluctuations much stronger than the ones from the network. This would mean that external inputs drown network interactions, effectively driving neurons into an “uncoupled” regime for which reliable responses are expected [51, 52]. For the parameters chosen here (*η* = −0.5, *ε* = 0.5) however, the input and network fluctuations have comparable strengths with *ε*/*σ*_*E*,*I*_ ≃ 1.1 (see Methods). Additionally, the greater proportion of reliable spikes from the network compared to the surrogate population indicates that some reliable spikes in a given neuron are generated when the network inputs to that neuron are repeated across trials. We verified that spike reliability is unchanged by network size *N*.

Finally, the fact that spike reliability is robust to moderate changes in connectivity in-degree *K* (Fig 5 (D)) is consistent with the scaling of fluctuation strengths *σ*_*E*,*I*_. Indeed, *σ*_*E*,*I*_ does not explicitly depend on *K* and is independent of *N* (see Eq (6) in Methods). The ratio *ε*/*σ*_*E*,*I*_ only changes with overall firing rates *ν*_*E*,*I*_, which vary slowly and monotonically with *K* (see Fig 4 (B)). This suggests that *N* should have little effect on the discrimination properties described in the rest of this paper, and that *K*’s only influence manifests through its modulation of *ν*_*E*,*I*_. Thus, we concentrate on networks of fixed size *N* = 500 and in degree *K* = 20. We chose these parameters both for ease of simulation and to demonstrate that even small networks can have rich coding properties while remaining in balanced regimes.

We summarize these findings in the following heuristic description. The response variability of our recurrent network is characterized by intermittent reliability, where some spikes are always reproduced across trials and others are not. Which spikes are reliable depends on the input and its history in non-trivial ways. In some cases, a spike is reliably elicited when the signal *I*_*i*_(*t*) has a sufficiently strong upswing, thus directly encoding a feature of a local input. However this is not the only way reliable spikes are produced; others occur when a neuron receives network input from other cells with coincident reliable spikes. Network interactions are themselves partially stimulus locked, but still show some cross-trial variability because of chaos. This has the effect of increasing the “fraction” of all inputs to a neuron that is frozen from trial to trial, compared to the naive mean field assumption, thus increasing reliability. It is this interplay between network and external inputs that create complex response statistics, a signature of which is the intermittent presence of reliable spikes.

Intriguingly, similar types of “intermittent” spiking variability have been reported in *in vivo* experiments (cf. [33, 34]). As we see below, this is best described by attributes of stimulus-dependent chaotic attractors, with low dimensionality, and occupying specific regions of state space. These network interactions are not easily captured by the kind of statistical ensembles usually used to derive MF equations, in which one considers system trajectories with random initial conditions *and* independent stimuli; the assumptions that are valid for such ensembles no longer hold for our response ensembles, which are predicated on a specific stimulus.

Thus, we conclude that *trial-to-trial variability in chaotic networks is more complex, and less severe than that of simplified stochastic models, leading to a great number of reliable spikes repeated across trials*. While it is conceivable that a stochastic model of network interactions can be derived to capture this phenomenon, it is not clear how to implement the various statistical dependencies outlined above. We show below that a state space view based on a dynamical systems approach is better suited to understand the mechanisms underlying this phenomenon.

### Chaotic attractors shape statistical dependencies across trials

To better understand the prevalence of reliable spikes in driven networks, and why this reliability appears to be intermittent (see Fig 5 (A)), we turn to a geometric view of network dynamics as captured by response ensembles. Below, we first review important concepts of input-driven chaos that were originally presented in [14, 15, 54]. In turn, we relate features of chaotic attractors to trial-to-trial variability and then to input-dependence, toward our goal of studying discriminability.

#### Chaos: A state space view of variability

Recall that the variability of a network’s response to *I*(*t*) is characterized by the breadth of differences between the trajectories forming its response ensemble Θ_*I*_, i.e., the “size” of Θ_*I*_ as measured by, e.g., its diameter. Although difficult to describe by simple mathematical formulae, for specific systems, response ensembles are well-defined mathematical objects with well-understood geometric properties that can be numerically characterized. The theory of random dynamical systems (RDS) provides a framework for studying these properties.

Snapshots of all trials in Θ_*I*_ at any time *t* > 0 are ensembles of points, corresponding to a probability distribution that describes all possible network states given that the system has been subjected to the stimulus *I*(*t*) up to time *t*. Taken together for all *t* > 0, these “snapshot distributions” [55] define input- and time-dependent probability distributions describing the network’s evoked activity for all possible initial states at once and they dictate statistical attributes of our network such as trial-to-trial variability. One of the key results of RDS theory is that under very general conditions, the ensembles Θ_*I*_ are concentrated on time-evolving geometric objects known as *random attractors*, so called because almost all initial conditions, when subjected to the same forcing *I*(*t*), will converge to the attractor. Unlike “classical attractors” in un-driven systems, which have fixed positions in space (e.g. fixed points, periodic orbits, strange attractors), the position of Θ_*I*_ changes in time *t* and with input choice *I*(*t*). This is because our networks are driven by time-dependent stimuli, and are governed by non-autonomous systems of differential equations (see Methods).

Geometrically, these random attractors can be points, curves, or higher-dimensional surfaces, and can wind around state space in complicated ways. The exact position and shape of an attractor depends on both the system parameters and the specific realization of the stimulus. Incidentally, this is why attractors are poorly modeled by models of stochastic noise that assume independence across dimensions, as demonstrated in the previous section. However, RDS theory also states that certain important properties of the attractor—and thus the response ensembles Θ_*I*_—are independent of specific choices of input *I*(*t*), are invariant in time, and depend only on system parameters. An important one is the sensitivity of network responses to small perturbations, as measured by the *Lyapunov exponents* of the system. For an *N*-dimensional system, these exponents are real numbers *λ*_1_ ≥ ⋯ ≥ *λ*_*N*_ that describe the rate of separation of nearby trajectories in different state space directions. For a system like our network, the Lyapunov exponents do not depend on the choice of input realization *I*(*t*) so long as it is a realization of white noise with same parameters *ε* and *η*. A criterion for chaos is the presence of positive Lyapunov exponents. Moreover, the number of positive Lyapunov exponents roughly indicates the number of unstable directions in state space, and their magnitude indicates how strong the amplification of small perturbations is in those directions. In a chaotic system, almost any nearby trajectories will diverge from each other exponentially fast, but they do so only along unstable directions of attractors.

Other geometric properties of random attractors can be related to their Lyapunov exponents as well. For example, the *attractor dimension* is a quantity describing how much of state space is occupied by the attractor—the source of trial-to-trial variability for our network—and is (roughly) given by the number of positive Lyapunov exponents. To see that this should be the case, imagine a cloud of initial conditions evolving according to the same stimulus. The state-space expansion associated with positive exponents tends to “stretch” this cloud, leading to the formation of smooth Λ^+^-dimensional surfaces, where Λ^+^ is the number of positive exponents (see, e.g., [44] for a general, non-technical introduction and [56, 57] for more details). If all exponents are negative, for example, then the attractor is just a (time-dependent) point, whereas the presence of a single positive exponent suggests that the attractor is curve-like, etc.

In previous work, we showed that in a wide variety of dynamical regimes, the number of positive Lyapunov exponents in driven balanced networks is less than 20% of the network’s dimension *N*, and often remains below 10% [14, 15]. In most of the paper below, we choose network and input parameters, described in *Methods*, so that about 8% of Lyapunov exponents are positive. Here, the rate of trajectory separation is strong with *λ*_1_ ≃ 3.5, and the network is by all accounts in a chaotic regime. At the same time, there are plenty of directions in which the attractor is “thin”, leading to trial-to-trial variability that is far from homogeneously random at the population level. We explore different parameter regimes, leading to distinct attractor dimensions, toward the end of this article.

#### Geometry of response ensembles and state-space separability

Our goal is to describe the geometry of chaotic attractors in state space sufficiently well so to explain the presence of reliable spike events described in the last section. Moreover, we use the same approach to ask about differences that arise between two attractors, generated by distinct inputs *I*^*A*^(*t*) and *I*^*B*^(*t*).

From the discussion above, we know that for two inputs *I*^*A*^(*t*) and *I*^*B*^(*t*) with identical statistics, the dimension of their associated response ensembles, and thus their level of trial-to-trial variability, will be the same. Revisiting our discrimination task, network responses will be discriminable if and only if the corresponding ensembles Θ_*I*^*A*^_ and Θ_*I*^*B*^_ do not overlap most of the time. To predict when this is the case, knowing only the dimension of Θ_*I*^*A*^_ and Θ_*I*^*B*^_ is insufficient; we need to understand how the position of Θ_*I*_ in state space depends on the choice of *I*(*t*). We will present evidence that for the balanced spiking networks studied here, there exist broad parameter regimes where the relation: “*diameter of a response ensemble* ≪ *distance between different ensembles*” generally holds.

**Fig 6:**
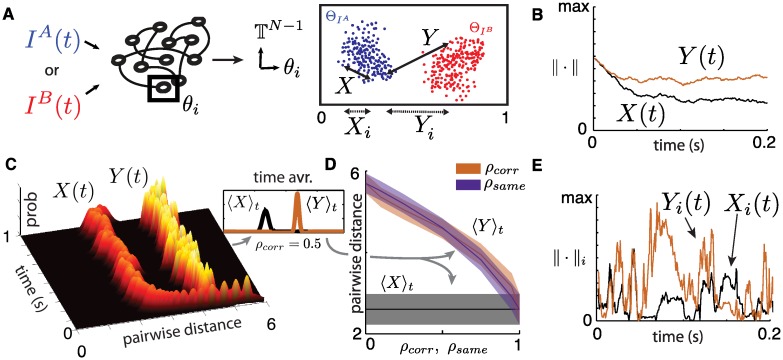
(**A**) Illustration of pairwise distances between trajectories within an ensemble (*X*) and across ensembles (*Y*). Also illustrated are equivalent distances for a single neuron’s state variable *θ*_*i*_ (*X*_*i*_, *Y*_*i*_). (**B**) Pairwise distances between two trajectories for the entire network (on the *N*-dimensional torus $T^{N}$, *N* = 500) as a function of time. (**C**) Time evolution of pairwise distance distributions for entire network (*N* = 500), sampled with 100 trials from each ensemble. After a very fast transient (∼ 10-50 ms), distributions settle into stable values. For panels (B,C,E), *ρ*_*corr*_ = 0.5. (**D**) Mean of stationary pairwise distances for a range of similarity parameters *ρ*_*corr*_ or *ρ*_*same*_ (independently modulated). Shaded areas show one standard deviation. Both mean and standard deviation were averaged over 1000 time points between *t* = 100 and *t* = 12,000 ms. (**E**) Pairwise distances between two trajectories in a single neuron’s coordinate *θ*_*i*_ as a function of time.

Consider the following pairwise distance quantities, the statistics of which we sample using numerically simulated network trajectories (see Methods for details):

$$\begin{array}{cc} X(t)= & ∥\theta \left(t,\theta_{0},I^{A}\right)-\theta \left(t,{\tilde{\theta }}_{0},I^{A}\right)∥\\ Y(t)= & ∥\theta \left(t,\theta_{0},I^{A}\right)-\theta \left(t,{\tilde{\theta }}_{0},I^{B}\right)∥ \end{array}$$ (9)

where *θ*(*t*, *θ*_0_, *I*) denotes the network trajectory in state space, given an initial state *θ*_0_ at *t* = 0 and subject to input stimulus *I*(*t*). Initial states *θ*_0_ and ${\tilde{\theta }}_{0}$ are independently, randomly chosen and ‖*θ*_1_ − *θ*_2_‖ denotes the (shortest) distance between two states *θ*_1_ and *θ*_2_. Fig 6 (A) illustrates these measurements. Both *X*(*t*) and *Y*(*t*) denote the distance between a pair of trajectories at time *t*: “within-ensemble” for *X*(*t*) and “between-ensemble” for *Y*(*t*). Formally, they are random variables because they depend on a pair of random initial conditions.

The quantity *X*(*t*) represents the “typical size” of an ensemble at time *t*; we view it like the diameter in that it measures the distance between two typical points on the corresponding attractor. The quantity *Y*(*t*) is the typical distance between two ensembles elicited by stimuli *I*^*A*^(*t*) and *I*^*B*^(*t*). In the context of the present model, we say that the two stimuli are *separable* (in the state space sense) if the distributions of *X*(*t*) and *Y*(*t*) do not have a significant overlap (see Fig 6). The dimension of the underlying attractor is reflected in *X*(*t*) in the following way: if the dimension were 0 (so the attractor is a single point), then *X*(*t*) ≈ 0, and if the dimension is positive, then *X*(*t*) would also be positive (but can be large or small). In general, *X*(*t*) will fluctuate in a complicated fashion as the time-dependent attractor evolves and changes shape. Based on previous work [14, 15], we expect these fluctuations to be relatively small, so that *X*(*t*) is nearly constant in time. Furthermore, while the fluctuations in *X*(*t*) depend on the specific choice of stimulus, standard results from probability theory tell us that its mean depends only on the system parameters and the statistical distribution of the stimuli. Thus, the mean value of *X*(*t*) remains unchanged if we replace *I*^*A*^(*t*) by *I*^*B*^(*t*) in Eq (9) (since we require *I*^*A*^(*t*) and *I*^*B*^(*t*) to have identical parameters). Similar reasoning applies to *Y*(*t*), which is akin to measuring the rate of separation between two independent realizations of the same stochastic process. Once again, we expect *Y*(*t*) to fluctuate around some mean, with the exact fluctuations dependent on the input realizations but with mean and variance depending only on system parameters and stimulus statistics.

From simulations, we produce pairs of trajectories subject to both *I*^*A*^(*t*) and *I*^*B*^(*t*), all with random initial states. We compute *X*(*t*) and *Y*(*t*) and plot the result as a function of time in Fig 6 (B) where *ρ*_*corr*_ = 0.5 (*I*^*A*^ and *I*^*B*^ are 50% correlated). As expected, after a short transient *X*(*t*) settles quickly to a positive constant, which is unchanged if the trajectory pair is selected from $\Theta_{I^{A}}^{t}$ or from $\Theta_{I^{B}}^{t}$. Likewise, *Y*(*t*) settles quickly to a steady-state in which it fluctuates around a well-defined mean.

For a more complete view of this phenomenon, we consider the distributions of pairwise distances, sampled over 100 trajectory pairs, starting from uniformly random states. Fig 6 (C) shows the time evolution of these distributions for the first second of elapsed time. As for the single-pair measurements (Fig 6 (B)), both distributions settle into near-constant, steady values after a very fast transient (∼10-50 ms). This remains true for any similarity parameter value *ρ*_*same*_, *ρ*_*corr*_. We note that this short transient validates our general definition of “trial” which includes trajectories with distinct initial states as well as any trajectories that received some sort of perturbation—such as a synaptic failure or the event of an extra spike—in the recent past. To capture the stationary nature of pairwise distances, we compute time-averaged distributions 〈*X*〉_*t*_ and 〈*Y*〉_*t*_, which we calculate using 1000 time points between *t* = 100 and *t* = 12,000 ms. While 〈*X*〉_*t*_ remains the same regardless of the similarity between *I*^*A*^(*t*) and *I*^*B*^(*t*), 〈*Y*〉_*t*_ can be used to measure the effect of stimulus similarity on the location of response ensembles in state space. Fig 6 (D) shows the means 〈*X*〉_*t*_ and 〈*Y*〉_*t*_ as well as one averaged standard deviation, for a range of input similarity parameters *ρ*_*corr*_ and *ρ*_*same*_ between 0 and 1. Here, only one similarity parameter is varied at a time, while the other is kept at zero. As expected, when both inputs are identical (*ρ*_*corr*_ = 1 or *ρ*_*same*_ = 1), 〈*Y*〉_*t*_ collapses to 〈*X*〉_*t*_ since Θ_*I*^*A*^_ and Θ_*I*^*B*^_ describe the same ensemble.

However, we see that 〈*X*〉_*t*_ and 〈*Y*〉_*t*_ become more than two standard deviations apart as soon as the stimuli become less than 90% similar. This is true for both definitions of stimulus similarity. The conclusion is that the chaotic, balanced networks at hand produce dynamical responses that stay separated in state space even for stimuli that are very similar.

Finally, the geometric attributes described above can be related to the reliability of spike times already discussed in the previous sections (see Fig 5). Previous work has shown that the unstable directions in attractors—i.e., the directions in which chaotic dynamics will spread the response ensemble produced by a single stimulus—generally align with neural coordinates. Moreover, the identity of the corresponding “unreliable” neurons change in time [14]. This leads to intermittent variability in single neurons: at any moment there is a small fraction of neurons in the network that have variable dynamics across trials, while the rest behave in a reliable fashion.

We can observe this phenomenon by restricting the definition of pairwise distance Eq (9) to single-neuron coordinates: *X*_*i*_(*t*), and *Y*_*i*_(*t*) for the (transformed) voltage variable *θ*_*i*_. The value *X*_*i*_(*t*) = 0 means that different initial network states nevertheless lead to the same state for cell *i* at time *t*, i.e., the neuron *i* has the same voltage across all trials. A similar interpretation applies to *Y*_*i*_(*t*). Fig 6 (E) shows these quantities for a randomly selected neuron *i*. In contrast to pairwise distances in the full state space (Fig 6 (B)), *X*_*i*_(*t*) regularly collapses to zero. This is a reflection of the intermittent spike event reliability discussed earlier. *Y*_*i*_(*t*) also varies over time although it remains greater on average. This suggests that at any moment in time, for a given input *I*(*t*), some network coordinates may offer trial-to-trial reliable responses that can be used to distinguish similar input stimuli. We investigate the use of evoked spike times for discriminability in the next section.

To summarize, pairwise distances between evoked trajectories inform us about geometric properties of response ensembles, and how they organize in state space. In parameter regimes where *X* ≪ *Y*, which occur for stimuli that are less than 90% similar, we expect that a decoder could classify novel trajectories, given information about the distributions 〈*X*〉_*t*_ and 〈*Y*〉_*t*_. Conversely, in parameter regimes where *X* ≈ *Y*, it may be difficult for a downstream decoder to discriminate response ensembles using simple criteria like linear separability. Moreover, the separation between trajectories within an ensemble remains constant in time but is supported by only a small fraction of neurons, the identity of which changes over time. This leads to intermitting spike-time reliability with statistics that have population-wide dependencies.

Thus, *the geometric attributes of chaotic attractors both enable separability of network responses evoked by distinct stimuli, even when the stimuli have similarities, and explain intermittent spike time reliability within a response ensemble*. We show below how a decoder can exploit these features to classify inputs.

### Finding 1: Chaotic spike patterns are linearly discriminable

The state-space separability studied above assumes that one has access to the full state of the network at all times; any biologically realistic decoder would only have access to a network’s spiking activity. We next present evidence that the *spikes* generated by balanced, chaotic networks can also be used by a simple linear classifier, the Tempotron (see Methods), to discriminate between two stimuli with identical statistical properties.

We observed that the Tempotron classification performance *P* roughly follows the trends found above for the pairwise distance between response ensembles (see Fig 6): there is perfect classification (*P* = 1) until input similarity reaches about 90% (i.e. *ρ*_*same*_, *ρ*_*corr*_ = 0.9). This means there exists a linear combination of evoked spike patterns that reliably sum to cross a threshold for only one of the two stimuli, regardless of network initial conditions. Thus, when distinct stimuli are presented to chaotic networks, even ones with very similar features, it is not only the network states they produce that are highly discriminable, but also the resulting spike trains. We will return to the question of performance versus input similarity below, but first turn to its mechanism.

We propose that reliable spiking of a few neurons at precise moments in time (i.e., reliable spike patterns) drive successful classification of stimuli by the Tempotron. In this section we demonstrate this by deconstructing the Tempotron’s readout and by observing the impact of “jittering” underlying spikes.

**Fig 7:**
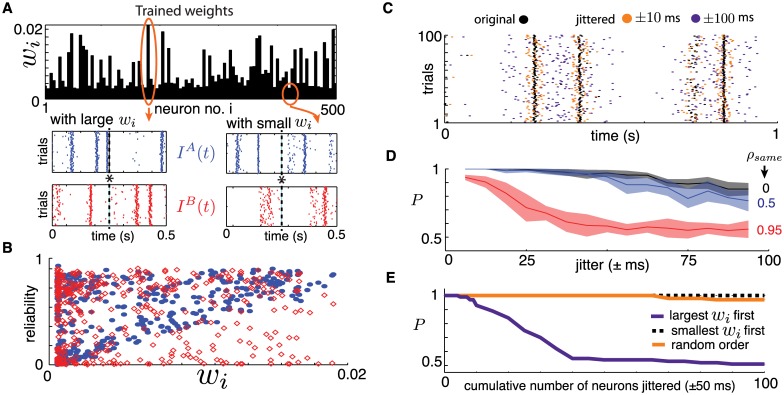
(**A**) Top: Trained Tempotron weights *w*_*i*_. Bottom: example cross-trial spiking output for neurons associated with large and small weights *w*_*i*_. Asterisk shows the time at which the maximal value of the Tempotron variable *V*(*t*) is obtained (see also Fig 3 (B)). (**B**) Spike-time reliability for the closest spike to the maximum (asterisk), plotted for each cell against the readout weight *w*_*i*_. Solid blue markers indicate responses to *I*^*A*^(*t*) and hollow red markers to *I*^*B*^(*t*). (**C**) Example raster plot for a single neuron across 100 trials, with and without spike-time jittering. Two jitter strengths shown: ±25 and ±100 ms. (**D**) The Tempotron’s performance *P* when all neurons are jittered, plotted against jitter strength, for three values of *ρ*_*same*_. The classifier was trained and tested on jittered spike time data. (**E**) Tempotron’s performance *P* against the cumulative inclusion of neurons where the spike times were strongly jittered (±50 milliseconds). Different lines indicate three orderings of neurons the neurons that were jittered, based on their trained readout weight *w*_*i*_: (i) decreasing, (ii) increasing and (iii) random. The classifier was trained on original data and tested on jittered data from a network with *N* = 500, *K* = 20. For all panels except (D), *ρ*_*corr*_ = *ρ*_*same*_ = 0.

We first turn to the readout weights *w*_*i*_, which are the result of a global optimizing algorithm [35]. Fig 7 (A) shows the values of all *w*_*i*_’s for a Tempotron trained to distinguish two stimuli, *I*^*A*^(*t*) and *I*^*B*^(*t*), with perfect performance (*P* = 1). We find that the neurons with the strongest weights spike very reliably when the network is presented with input *I*^*A*^(*t*), right before the peak of the Tempotron’s output (asterisk, see Fig 7 (A) bottom and Fig 3 (B)). Conversely, the same neurons either do not spike or spike unreliably in response to *I*^*B*^(*t*) around the same moment in time. In Fig 7 (B), we quantify this finding by showing a scatter plot of spiking event reliability for each neuron, plotted against its output weight *w*_*i*_. This shows that any neuron with a high *w*_*i*_ also shows high spiking reliability under input *I*^*A*^(*t*).

Next, we degrade the temporal precision of network responses by randomly “jittering” spike times and study the impact on classification performance by the Tempotron. To “jitter” a spike train, we shift each spike time on all trials by a random amount, uniformly and independently drawn from an interval of (−*r*, *r*), where *r* is the *jitter radius* (see Fig 7 (C)). This leaves the total number of spikes fired the same, but strongly disrupts their temporal precision, as illustrated in Fig 7 (C). We use this jittering in two ways. First, we train and test the Tempotron on jittered spikes, to probe the dependence of classification performance on the temporal precision of spike times produced by chaotic networks. Second, we train the Tempotron on the original spike data but test using jittered spike times for subsets of neurons, to probe the learned role of these neurons in classifying stimuli.

Training and testing the Tempotron on jittered spike time data shows that classification performance *P* progressively declines as the jitter radius increases. The rate at which performance drops depends on the similarity between inputs *I*^*A*^(*t*) and *I*^*B*^(*t*), as illustrated in Fig 7 (D) for three values of *ρ*_*same*_ (the fraction of neurons receiving identical direct inputs under both signals). Evidently, the lower *ρ*_*same*_ is, the more distinguishing features there will be in the two response ensembles to be classified, the combination of which enables the Tempotron to classify stimuli even with substantial spike time jitter. Overall, when spikes are jittered by 10’s of ms, classification performance drops significantly; for similar stimuli, performance drops halfway to chance when the jitter radius is 25 ms. We conclude that the Tempotron uses quite precisely timed spikes to distinguish the responses of chaotic networks to nearby stimuli.

To probe the question of what spiking features lead to the reliable classification learned by the Tempotron, we set the jitter radius to 50 ms and jitter different subsets of neurons in the testing data. In contrast to the procedure described above, where training and testing spike times were jittered, only testing spike times are now jittered; the Tempotron is trained on the original spikes produced by the network. We apply this jittering procedure to an increasing number of neurons in the network, re-testing the classification performance *P* as neurons are cumulatively added to this “jittered” pool. We do this for three different orderings in which neurons are added to the jittered pool: (i) neurons with largest trained weights *w*_*i*_ listed first, (ii) neurons with smallest *w*_*i*_’s listed first and (iii) neurons randomly listed. The results are shown in Fig 7 (E). First, note that perturbing the spike times of neurons with large *w*_*i*_’s quickly reduces performance down to chance, whereas jittering the spike times of random neurons or those with low readout weights has little to no effect on performance. The details of the observations above are likely to depend on the choice of time constants for the Tempotron’s filters, but we expect the overall trends to persist for a range of these constants, based on the spike-time reliability described earlier. From this we conclude that the Tempotron learns to classify stimuli based on the reliable spikes of a few neurons, at precise moments in time.

Finally, we shuffled the spike ensembles across trials by building surrogate spike patterns in which the spike output of each neuron is taken from a randomly chosen trial from the ensemble as was originally done in [14, 15]. If the readout cells that serve as strong inputs to the Tempotron (i.e., the cells with relatively large *w*_*i*_) were unreliable, this would have the effect of shifting their spike times and changing the spike counts within the test window. Numerical results indicate that shuffling has no appreciable effect on classification performance. This further suggests that repeatable spike patterns are responsible for good classification, rather than statistics like spike counts on longer time scales.

Taken together, these tests show that *despite chaos, the network response preserves a fair degree of spike reliability across trials in key neural coordinates, and a simple decoding scheme is capable of taking advantage of this reliability to accurately classify spike trains.* Note that the identity of these “key” neurons will change for different input stimuli and thus, at least in principle, many readout schemes could be trained in parallel on the same network.

### Finding 2: Recurrence enables information distribution within networks and discrimination using few readouts

We have shown that a neural-like readout, the Tempotron, can use the reliable spikes embedded in a chaotic network’s response in order to classify input stimuli. Up to now, this classifier had access to spikes from every neuron. A natural question is whether this complete access is necessary. In other words, how does classification performance depend on the number of inputs and outputs to the network? For example, if only a few neurons in the network receive discriminable inputs ($I_{i}^{A}\left(t\right)\neq I_{i}^{B}\left(t\right)$) and the decoder only reads out from a few (different) neurons, do network interactions distribute enough stimulus information to enable a successful classification?

**Fig 8:**
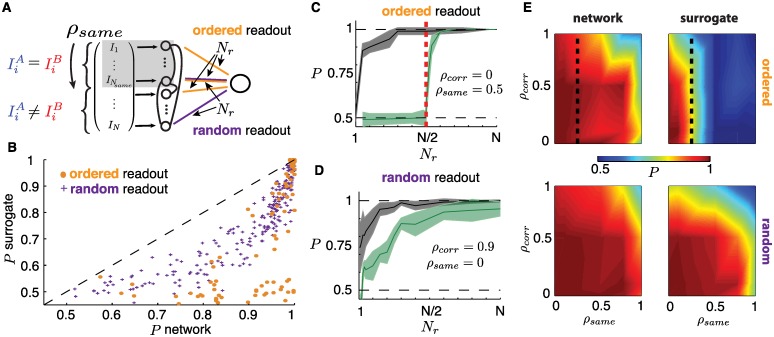
(**A**) Illustration of input similarity *ρ*_*same*_ for which the first *N*_*same*_ neurons receive identical direct inputs $I_{i}^{A}\left(t\right)$ and $I_{I}^{B}\left(t\right)$. Also depicted is partial readout dimension *N*_*r*_ under two types of readout orderings: *ordered* and *random*, see text for details. (**B**) Scatter plot of classification performance *P* of surrogate population vs. network for a range of parameters *ρ*_*corr*_, *ρ*_*same*_ and *N*_*r*_, for both ordered and random readouts (same data as (E)). (**C**) *P* for network (black) and surrogate population (green) as a function of *N*_*r*_ with ordered readouts, for *ρ*_*corr*_ = 0, *ρ*_*same*_ = 0.5. Red dashed line indicates *N*_*r*_ = *N*_*same*_. (**D**) *P* for network (black) and surrogate population (green) as a function of *N*_*r*_ with random readout, for *ρ*_*corr*_ = 0.9, *ρ*_*same*_ = 0. For (C) and (D), shaded areas show one one standard deviation (see sampling details below). (**E**) Color plot of *P* as a function of *ρ*_*same*_ and *ρ*_*corr*_ for *N*_*r*_ = *N*/4. Top row: ordered readouts (dashed line indicates *N*_*r*_ = *N*_*same*_). Bottom row: random readouts. First column: network. Second column: surrogate population. For all *P* values shown, quantities were sampled over 20 repeated simulations where networks, inputs and Tempotron training were reprocessed. *N* = 500, *K* = 20.

In our model, the proportion of the input that is directly discriminable is controlled by the parameter *ρ*_*same*_—the fraction of cells that receive identical inputs under *I*^*A*^(*t*) and *I*^*B*^(*t*). We adopt an enumeration that lists these cells first for ease of notation: the first *N*_*same*_ neurons receive indistinguishable inputs while the remaining *N*_*diff*_ receive independent ones for each stimulus; with *N*_*same*_ = *ρ*_*same*_ *N* and *N*_*diff*_ = *N* − *N*_*same*_ (see Fig 2 (B)). In addition, we introduce a second parameter, *N*_*r*_, which controls the number of readout neurons providing inputs to the Tempotron. We select these *N*_*r*_ cells in two ways as depicted in Fig 8 (A): *ordered readouts* where we follow the same ordering as above (i.e. the Tempotron weights *w*_*i*_ are defined for indices *i* = 1, …, *N*_*r*_) and *random readouts* where *N*_*r*_ neurons are selected at random throughout the network.

We investigate the classification performance *P* of our network with different configurations of input similarity, readout size and type. For example, if *ρ*_*same*_ = 0.5 (equivalently *N*_*same*_ = *N*/2), *ρ*_*corr*_ = 0.75 and *N*_*r*_ = *N*/4 with ordered readouts, this means that neurons *i* = 1, …, *N*/2 receive identical local inputs $I_{i}^{A}\left(t\right)$ and $I_{i}^{B}\left(t\right)$, the remaining neurons *i* = *N*/2 + 1, …, *N* receive local inputs that are 75% correlated, and the Tempotron only reads out from neurons *i* = 1, …, *N*/4. In addition, for all parameter configurations, we also produce surrogate population spike patterns where we replace network interactions by independent Poisson-distributed spike trains as was done in Fig 5 (B), following the assumptions from earlier. The comparison is summarized in Fig 8 (B) where a scatter plot of surrogate performances is plotted against network performances for an exhaustive range of parameter choices. Importantly, the chaotic network always performs better or as well as the surrogate population. Furthermore, the only cases in which the two perform equally is when the discrimination task is easy enough to allow perfect performance.

The scatter also shows that the network has a significant advantage over the surrogate population when ordered readouts are used. To better understand this, Fig 8 (C) shows network *P* (black) and surrogate *P* (green) for *ρ*_*corr*_ = 0, *ρ*_*same*_ = 0.5 as a function of *N*_*r*_ for ordered readouts. Notably, the network shows very high classification performance in many cases where *N*_*r*_ < *N*_*same*_. Here the classifier can *only* read out from neurons that receive identical inputs under the two stimuli (i.e. not directly discriminable). This means that discriminable features of these neurons’ spike patterns cannot originate from their direct inputs $I_{i}^{A}\left(t\right)$ and $I_{i}^{B}\left(t\right)$, and must instead result from network interactions that communicate activity from other neurons. Not surprisingly, the surrogate population fails completely when *N*_*r*_ < *N*_*same*_ since network interactions are replaced by independent stochastic spike trains.

For random readouts, it can be assumed that at least one neuron selected for readout receives a directly discriminable inputs when *ρ*_*same*_ < 1. This makes the task easier so that network and surrogate population performances are closer when local inputs *I*_*i*_(*t*) are sufficiently different. Nevertheless, when the correlation between them grows, i.e. for larger *ρ*_*corr*_, the network does produce significantly more discriminable responses. This is shown in Fig 8 (D) where *P* is plotted as a function of *N*_*r*_ for *ρ*_*same*_ = 0, *ρ*_*corr*_ = 0.9 and random readouts.

The general landscapes of discriminability *P* as a function of similarity parameters *ρ*_*corr*_ and *ρ*_*same*_, for both ordered and random readouts, are shown in Fig 8 (E) for *N*_*r*_ = *N*/4. Consistent with the results about the geometric properties of response ensembles shown in Fig 6 (D), we achieve near-perfect performance (*P* ≈ 1) up to *ρ*_*same*_ ∼ 0.9, despite reading out from only a quarter of the network’s neurons. Crucially, we see that the phenomenon introduced in panel (C) of the same figure—that network can accurately discriminate inputs when reading out from neurons not receiving directly discriminable inputs—is robust to strong input correlations *ρ*_*corr*_.

We conclude from these results that *recurrent interactions of the type found in our network model, despite generating chaotic instabilities, are an effective and robust way to distribute information about local inputs throughout a network so that distant readouts can be used for stimulus discrimination without specifying precise connectivity.* We expect these findings to hold generally in recurrent networks that are well-connected, in the sense that for every pair of vertices in the associated connectivity graph, there is a relatively short directed path between them. In the next section, we show how the relationship between the number of readouts *N*_*r*_ needed for classification and the number of distinct inputs *N*_*diff*_ depends on stimulus statistics.

### Finding 3: Signal strength modulates a “noise floor” from chaos

So far, our investigation of stimulus encoding has been restricted to a single choice of the stimulus amplitude *ε* and mean *η* (*ε* = 0.5, *η* = −0.5). For these parameters, we showed that even highly similar stimuli can be distinguished based on the responses of chaotic networks. How does this depend on the stimulus amplitude (i.e. strength of temporal features)? When this amplitude drops, one might expect that it will eventually fall below a limit when any differences in stimuli will be obscured by variability induced by the chaotic dynamics of a network. We call this limit the chaos-induced *noise floor*. Below, we study this noise floor, and thereby establish how the discriminability of stimuli in chaotic networks depends on their statistics.

**Fig 9:**
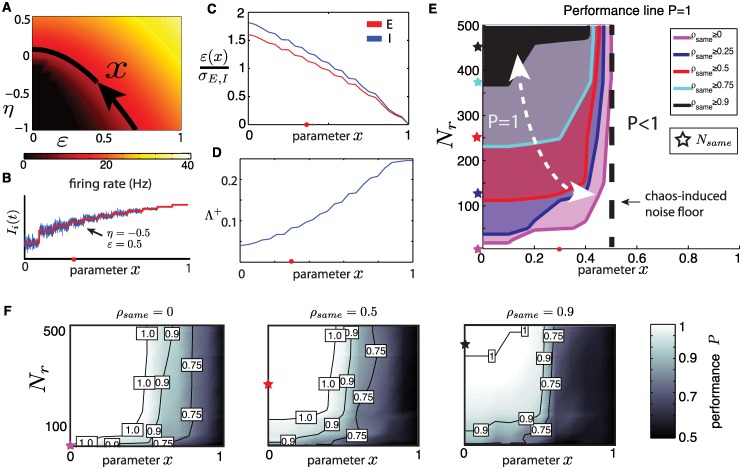
(**A**) Mean E firing rate of the network as a function of mean input *η* and signal amplitude *ε*. Black curve shows level set at 13 Hz, and is parametrized by the normalized arclength *x* in direction of arrow. Parameter set used throughout the rest of paper (*ε* = 0.5, *η* = −0.5) corresponds to *x* ≃ 0.3. (**B**) Illustration of input *I*_*i*_(*t*) presented to a neuron as a function of the parameter *x*. The red line shows the mean *η*(*x*) while the blue line shows fluctuations of amplitude *ε*(*x*). For all parameters, the mean excitatory firing rate is constant. Red dot shows the benchmark regime (*η* = −0.5, *ε* = 0.5). (**C**) Ratio of stimulus amplitude *ε* to that of network interaction amplitude *σ*_E,I_ as derived from the mean field equations (see Eq (6)). Red dot shows the benchmark regime (*η* = −0.5, *ε* = 0.5). (**D**) The fraction of positive Lyapunov exponents #{*λ*_*i*_ > 0}/*N* as a function of the parameter *x*. Red dot shows the benchmark regime (*η* = −0.5, *ε* = 0.5). (**E**) Level curves of classification performance *P* = 1 in the (*x*, *N*_*r*_) parameter space for several values of *ρ*_*same*_. For parameter pairs to the left of these curves, the network achieves perfect classification on average. Stars indicate the corresponding *N*_*same*_ = *ρ*_*same*_ *N*. Red dot shows the benchmark regime (*η* = −0.5, *ε* = 0.5). (**F**) Classification performance *P* as a function of *x* and readout dimension *N*_*r*_ for three input similarity fractions *ρ*_*same*_ = 0, 0.5, 0.9. Stars indicate the corresponding *N*_*same*_ = *ρ*_*same*_ *N*. For all *P* values shown, quantities were sampled over 20 repeated simulations where networks, inputs and Tempotron training were re-processed. Ordered readouts, *ρ*_*corr*_ = 0, *N* = 500, *K* = 20.

To systematically compare how stimulus statistics impact discriminability, care is needed to keep the network activity in a fairly consistent firing regime. We do this by varying parameters (*ε*, *η*) together in a way that will produce a fixed firing rate, averaged across the network. This way, we can be certain that classifiers will be trained on the same number of spikes on average, a quantity that could affect the interpretation performance *P* if left uncontrolled. Specifically, we vary *η* and *ε* together along a path that leaves the network-wide average E firing rate fixed at 13 Hz, as was the case for parameter values used above (see Fig 4 (B)). As illustrated in Fig 9 (A), we parameterize this path by a normalized arclength parameter *x*: for higher values of *x*, *η* is larger and *ε* is smaller (see Fig 9 (B) for illustration of input as *x* changes).

We begin by visualizing the relative amplitudes of external inputs *I*_*i*_(*t*) and network interactions. If the strength of stimulus fluctuations *ε* was so great as to simply overwhelm interactions between neurons in the network, then the role of chaos in any conclusion about stimulus encoding would be trivial: the stimulus simply overwrites any intrinsic dynamics. Fig 9 (C) shows the ratio of stimulus input amplitude *ε* to that of network interactions *σ*_E,I_ computed from Eq (6). We can see that for the range of our parameter *x* this ratio goes from 0 to 1.8. Not surprisingly, the highest value of that ratio corresponds to the maximal value of *ε*, when *x* = 0. Even for ratios greater than 1 we argue that our network is not completely overwhelmed by inputs since for all parameters considered, our network is still chaotic with positive Lyapunov exponents (see Fig 9 (D) and text below). This means that on repeated trials, network interactions are strong enough to induce instabilities and create discrepancies between responses. However, these discrepancies will be smaller than in networks where input amplitude *ε* is weaker, impacting discriminability. This is what we investigate below. See also [14] for further investigation of this mechanism using spike-triggered averages.

Even if the stimulus input does not completely overwhelm the network, its statistics play an important role in shaping the level of discriminable features carried by network spike patterns. As we vary input parameters, two key quantities change. One is the “signal strength:” as *ε* becomes smaller, the direct impact of stimulus fluctuations on neurons subsides, and, as a consequence, so does the magnitude of the differences between stimuli *I*^*A*^(*t*) and *I*^*B*^(*t*). The second is the “noise:” the chaotic variability in the responses to a single input signal. These two quantities are not independent, and their relationship is not *a priori* obvious. Together, they combine to create the chaos-induced noise floor described above. For stimulus parameters that fall below this noise floor, chaotic variability is too widespread to allow different stimuli to be accurately classified.

As described earlier, the variability across spike patterns from the same response ensemble depends on the dimension of the underlying chaotic attractor. There are many ways to quantify this dimensionality, here we use the number of positive Lyapunov exponents divided by the dimension of the system (see Methods or [14] for details of their computation):

$$\Lambda^{+}\equiv \#\left\{\lambda_{i}>0\right\}/N.$$

Intuitively, Λ^+^ indicates the fraction of unstable directions in state space at any given time. As the geometric properties of our network attractors impose that those directions generally align with neural coordinates *θ*_*i*_ (see earlier text about spike-time reliability), Λ^+^ dictates how many neurons are “unreliable” in the system at any given time [14, 15]. Fig 9 (D) shows Λ^+^ as a function of *x*. Notice that the network becomes more chaotic (more positive Lyapunov exponents) as the fluctuation amplitude *ε* of the inputs *I*_*i*_(*t*) shrinks. This can be interpreted as weaker stimulus fluctuations giving less entrainment of neural dynamics by the inputs, and hence allowing intrinsic dynamics, the mechanism by which chaos emerges, to dominate (c.f. [31] for an example of stimulus entrainment effects on chaos in networks of rate units). Thus, as *x* increases, both signal and noise factors should conspire to make input stimuli less discriminable. We next quantify this effect, and study how it depends on input similarity (quantified by *ρ*_*same*_) and readout dimension *N*_*r*_.

We numerically estimate the Tempotron’s discrimination performance *P* along the *x*-parametrized curve of stimulus parameters, for a range of *ρ*_*same*_ and *N*_*r*_ values. We concentrate on regimes where *ρ*_*corr*_ = 0 and consider only ordered readouts, in order to better investigate the phenomenon of information distribution throughout the network described in the previous section. This determines regions of the parameter space (*x*, *N*_*r*_, *ρ*_*same*_) where perfect classification performance, *P* = 1, is achieved. The boundaries of these regions are shown in Fig 9 (E), where every parameter point to the left of the boundaries yields perfect classification. These boundaries have the following interpretation: for a given input parameters specified by *x*, one needs to readout from a number of neurons *N*_*r*_ greater than a given *ρ*_*same*_-boundary to be achieve perfect discrimination of inputs with a similarity level given by *ρ*_*same*_. As expected, the regions of perfect performance shrink as inputs become more similar. This means that more readout dimensions *N*_*r*_ are needed to discriminate more-similar inputs. Importantly, the boundaries are positioned at much lower *N*_*r*_ than the corresponding *N*_*same*_ = *ρ*_*same*_ *N* for all cases, indicating that networks across a broad parameter range can classify inputs using neurons that themselves do not receive discriminable inputs (i.e. $I_{i}^{A}\left(t\right)=I_{i}^{B}\left(t\right)$) as demonstrated for our benchmark parameter set in the previous section.

Moreover, there is a critical region of stimulus statistics (*x* ∼ 0.5) where all classification boundaries aggregate for high *N*_*r*_. This represents the chaotic noise floor for *x*, beyond which inputs cannot be perfectly discriminated. Fig 9 (F) illustrates this noise floor in more detail, by showing contour plots of classification performance *P* in (*x*, *N*_*r*_)-space for three values of *ρ*_*same*_. It shows that while *P* eventually drops to chance (0.5) as *x* → 1—an expected behavior since *I*^*A*^(*t*) and *I*^*B*^(*t*) become indistinguishable when *ε* = 0—this transition becomes sharper for higher input similarity *ρ*_*same*_. This suggests that when two stimuli have many identical components (high *ρ*_*same*_), the network can either classify them very well or not at all, depending on the stimulus amplitude. When inputs are significantly dissimilar, this transition is more gradual. Importantly, for large enough readout size *N*_*r*_, the noise floor is almost identical for all input similarities, indicating that for this network, perfect classification becomes impossible for any input similarity once Λ^+^ reaches about 11% of *N*. Lastly, we note that the noise floor boundary is roughly aligned with input parameters yielding a ratio of stimulus-to-network fluctuation amplitude *ε*/*σ*_*E*_ ∼ 0.9. This means that input stimuli need to be comparable or stronger than other network interactions to achieve discriminability.

In summary, for the chaotic networks at hand, *perfect stimulus classification can be achieved for a wide range of stimulus statistics and similarities, and classification can be achieved using spikes from relatively few readout neurons. However, once the stimulus amplitude falls below a chaos-induced noise floor, classification performance degrades rapidly.*

## Discussion

### Summary

Sparse, strongly connected recurrent neural networks used to model cortical activity in the brain often produce a *balanced state*, leading to chaotic dynamics [2]. We showed that despite asynchronous dynamics—where neurons have temporally de-correlated activity—chaos-induced variability in driven networks is not easily approximated by simple stochastic processes (e.g. using a mean-field approach). Instead, we found complex statistical dependencies across network spike outputs conditioned on a given input, i.e. across trials. We studied how this chaos—viewed as an intrinsic source of variability—impacts the capacity of recurrent networks to accurately encode temporal stimuli. With detailed numerical simulations grounded in the theoretical literature, we studied how similar stimuli—modelled by multi-dimensional frozen white noise inputs—can be decoded from chaotic network responses, both at the level of the network state space and output spike trains.

Two factors influence the ability of a decoder to successfully classify stimuli based on network outputs. The first is the strength of the chaos-induced “noise”: the trial-to-trial variability of evoked patterns due to chaos. The second is the “signal”: the sensitivity of evoked patterns to the choice of input. Our analysis of these separate factors leads to three main points: (1) Chaos in recurrent spiking networks does not, in and of itself, preclude the accurate encoding of temporal stimuli; simple decoders read out these stimuli based on reliable multi-spike patterns that chaotic networks produce via low-dimensional attractors. (2) Recurrent connectivity distributes stimulus information throughout chaotic networks, enabling high-dimensional stimuli to be classified with low-dimensional readouts. (3) Stimulus statistics (i.e., the amplitude of stimulus fluctuations relative to their mean) modulate the number of readout neurons necessary to successfully classify them: as their amplitude decreases, more neurons are required to discriminate stimuli, until a “noise floor” is reached where discrimination is no longer possible.

### Biological implications of encoding and computing in chaotic networks

Chaotic dynamics appear as an emergent property of recurrent connectivity between neurons [2, 6, 12, 31, 58, 59] that would be otherwise very stable and reliable (see [51, 52] and [42, 60] for reliability of single neurons). It is conceivable that such chaos is a significant contributor to experimentally observed variability as well (e.g. [17, 23, 61]). In this way, chaos amplifies and adds to other stochastic noise sources in biological networks. Moreover, we reiterate that the type of “intermittent spiking reliability” that is produced by chaotic dynamics is also observed experimentally *in vivo* [33, 34]. Although not direct evidence that chaotic dynamics is indeed present in recurrent neuronal networks in the brain, these observations are consistent with this hypothesis.

Beyond contributing variability and noise, there is a substantial literature addressing the potential advantages of chaotic dynamics for encoding and memory [62–64]. In many cases, chaotic networks act as “reservoirs” and synaptic connections are trained to use their activity to perform a given task. Here, we take a more pragmatic perspective, studying how chaotic networks work as “channels” that receive inputs and produce spike outputs that carry usable information. Furthermore, we show that the same recurrent connectivity that produces chaos also serves to distribute stimulus information throughout the network: discriminability is maintained even if a decoder only has access to small subpopulations of neurons, and even when the inputs to be discriminated do not directly drive the subpopulations. As such, recurrence may serve to simplify the process of reading out stimuli from large populations, eliminating the need for precise wiring—with the resulting chaotic dynamics being a manageable by-product. While this type of stimulus “spread” can also occur in multilayer feed-forward networks with fan-out between layers, a recurrent architecture does the same operation locally, without requiring that decoders be located downstream. We speculate that this mechanism may also be relevant for contextual coding [65, 66], where the response of some neurons to a fixed local input changes if a secondary contextual input to others differs. This role for recurrence complements many other functions that it may serve in neuronal computation (e.g., maintaining working memory, enabling winner-take-all computation, sharpening tuning curves, etc.).

Finally, we argue that the encoding mechanism based on spike patterns we outline in this paper enhances earlier balanced network encoding mechanisms. Classic results point to important properties of balanced population-averaged activity: its response to global external inputs is both rapid—much faster than single neuron time constants—and linear [5]. If the inputs to our network evoke different population firing rates, then population averages carry the necessary information for discrimination. In contrast, when two inputs have similar statistics and differ only in the fine temporal patterns they carry, we show that the same network can rely on spike-time based mechanisms to classify them. It is unclear if such dynamics are present in cortical circuits and if so, in which regime they typically operate. However, there is evidence of different activity states a given cortical network can take (e.g. up and down states) depending on various contextual factors [67]. In light of the results we outlined, it is conceivable that cortical networks encode different aspect of inputs depending on these input features. Under this assumption, the emergent nature of chaos in recurrent networks may act as a natural mechanism to implement adaptive coding schemes, without any changes required to the network or neurons themselves.

### Future work

The results presented in this manuscript address a specific class of models, albeit one that is fairly prototypical. Further studies should focus on the effect of single neuron dynamics and connectivity statistics on stimulus encoding. Specifically, while we briefly investigated the effect of modest variations of network size *N* and connectivity in-degree *K*, a more substantial study is needed to understand network behavior in large-size limits. Moreover, beyond the amplitude effects studied here, the correlation of stimulus inputs across neurons can also impact the resulting chaotic network responses (see Discussion of [14]). At the same time, these input correlations effectively diminish the dimensionality of the stimulus by introducing redundancies. An interesting area of future work is to better understand the relationship between input and output dimension with respect to stimulus coding in recurrent, spiking networks.

In experiments, is not an easy task to test whether or not a particular neural circuit is chaotic. Indeed, even for a dynamical system that does not receive input drive, and for which one can observe all degrees of freedom, it is still a hard problem to attribute variability to stochastic or deterministic (chaotic) mechanisms (see e.g. [68, 69]). Therefore, the problem of experimentally verifying the nature of variability in neural circuits found in the brain is not a simple one. Nevertheless, we note that some *in vivo* experiments show stimulus-evoked spikes that appear to have the type of intermittent variability we described in this article [17, 33]. This invites future work to make closer connections between mechanistic models of chaotic dynamics and neural recordings.
